# Risk Factors and Outcomes of Redo Craniotomy: A Tertiary Care Center Analysis

**DOI:** 10.7759/cureus.21440

**Published:** 2022-01-20

**Authors:** Muhammad Faraz Raghib, Muhammad Usman Khalid, Noor Malik, Mir Ibrahim Sajid, Umm E Hani Abdullah, Asra Tanwir, Syed Ather Enam

**Affiliations:** 1 Department of Surgery, Aga Khan University Hospital, Karachi, PAK

**Keywords:** brain tumors cns tumors, subdural hemorrhage, craniotomy, emergent neurosurgery, low and middle country (lmic)

## Abstract

Background and aim

Reoperation rate is defined as the percentage of patients returning to the operating room (OR) within 30 days of an initial craniotomy and undergoing a repeat (redo) craniotomy procedure. It is a key factor of quality-of-care assessments and has implications for outcomes, especially in oncological cases. Redo craniotomies are associated with improvement in neurological status and decreased mortality rate compared to non-surgical interventions but are associated with higher costs and risk of complications. It is important to gauge the indications and frequency of redo craniotomies as an index of quality of healthcare to improve patient outcomes. This study aimed to identify the indications, frequency, and outcomes of reoperation following an initial craniotomy in neurosurgical patients at a tertiary care hospital.

Methods

This retrospective cohort study was conducted at a tertiary care center in Pakistan and included all patients who underwent unplanned reoperation within 30 days of initial craniotomy from January 1, 2010, to December 31, 2017. Demographics, indications for index surgery as well as reoperation, and outcomes in the form of complications, neurological status, and mortality were collected from medical charts and analyzed.

Results

The study comprised 111 patients who underwent reoperations. Median age of the patients was 36 years (interquartile range {IQR}: 33 years). From a total of more than 1900 annual cases, the frequency of unplanned reoperations was 3.5%. The most common indication of unplanned reoperation based on MRI/CT was hemorrhage (40%, subdural hemorrhage was most common), followed by hydrocephalus (22%), cerebral edema (13%), and residual tumor (13%). The most common clinical reason for unplanned reoperation was a drop in Glasgow Coma Scale (GCS) (59%), whereas anisocoria was seen in 10.8% of patients. The highest mortality rate was observed in patients who were reoperated from post-operative day two to post-operative day seven (56%). Hypertension (p=0.014) and thrombocytopenia (p<0.001) showed significant associations with developing intracranial hemorrhage. Seventy-eight percent of patients showed significant improvement in their Karnofsky Performance Score (KPS) whereas 22% showed deterioration in their KPS.

Conclusion

The delivery of consistent quality healthcare relies on early detection and intervention in at-risk patients. Our center’s reoperation rate is consistent with the average range among other centers globally. Hypertension, anticoagulation, and antiplatelet therapy were common risk factors for redo craniotomies within 30 days. Patients with these conditions need special care to prevent returns to the operating room. Patients also need to be monitored for hemorrhage in the short term (one to two days) and hydrocephalus in the long term (two to 30 days) to intervene early if needed.

## Introduction

Surgical outcomes and their rates of complications are some of the most important factors to consider when choosing one surgical approach over the other. Neurosurgery, one of the more complex surgical disciplines, has higher overall morbidity and mortality rates than many other surgical fields [1,2]. A common platform to gauge the quality of care in surgical patients is the National Surgical Quality Improvement Program (NSQIP) which is nationally validated and has over 700 participating hospitals in the United States [3]. NSQIP data indicates that the most common complications of craniotomy include pneumonia, surgical site infections, and return to the operating room (OR) [4]. In the past, several quality indicators have been discussed some of which are readmission and reoperation rates, the rates of nosocomial and surgical site infections, and overall length of stay. These indicators, while easy to register, often overlook indication and subspecialty-specific issues.

Reoperation rate is defined as the percentage of patients returning to the operating room (OR) within 30 days of an initial craniotomy and undergoing a repeat (redo) craniotomy procedure. The term has gained popularity as a quality indicator of surgical procedures [5]. Some of the common indications for reoperation following craniotomy are post-operative bleeding, incomplete tumor resection, post-operative elevated intracranial pressure, shunt failure, superficial or intracranial surgical site infections, and post-operative cerebrospinal fluid (CSF) leak [2]. Furthermore, other factors which are associated with an increased reoperation rate include thrombocytopenia, hypertension, emergent surgery, long intraoperative time, dependent functional status, and morbid obesity [2-6].

Craniotomy, as with other surgeries, has its own share of potential complications which can lead to neurological (8.5%), regional (3-4%), and systemic (2-5%) symptoms [2]. The most common neurological symptoms arising from these complications include aphasia, dysphasia, visual field deficits, sensory deficits, and motor deficits. Regional deficits include hydrocephalus, pneumocephalus, CSF leak, wound infection, meningitis, and hematomas (epidural, subdural, and surgical cavity). Systemic signs include urinary tract infection, deep venous thrombosis, pulmonary embolism, sepsis, and psychosis. The mortality rate following craniotomy is 1.2% and the morbidity rate is around 8-12% [2].

Reoperation rate, as it relates to neurosurgery, has not been comprehensively discussed in the literature so far although it has been hypothesized to be a feasible parameter to evaluate the outcome. Studies have thus far focused more on an early reoperation rate within seven days or have evaluated pediatric populations. As evidenced by literature on other surgical disciplines, the 30-day reoperation rate could serve as a reliable indicator for the measurement of healthcare quality in neurosurgical patients [6]. In general, surgical procedures have a reoperation rate between 0.6% and 9.4% [7]. The average rate of reoperation after an initial craniotomy is around 4.8%; however, there are very little data for reoperation rates after craniotomies in Pakistan and other lower-middle-income countries (LMICs) [5]. In this study, the authors aimed to identify the indications, frequency, and outcomes of reoperation following an initial craniotomy in neurosurgical patients at a tertiary care hospital.

## Materials and methods

This retrospective study included patients who underwent surgical procedures at the Aga Khan University Hospital (AKUH), Pakistan, between January 1, 2010, and December 31, 2017. The Aga Khan University is a tertiary care hospital in the largest city of Pakistan and serves patients from Pakistan and surrounding countries. It is the first Joint Commission International (JCI) accredited hospital within the country. Our cohort included all patients who underwent an unplanned reoperation within 30 days of initial craniotomy. Patients who did not undergo reoperation or underwent reoperation later than 30 days were excluded from the study. The International Statistical Classification of Diseases and Related Health Problems-9 (ICD-9) code was used to retrieve a list of patients who underwent reoperations which numbered 166 individuals, and a total of 111 patients were isolated as per the selection criteria. Data were collected from online records and physical files maintained by the Health and Information Management System (HIMS) at AKUH.

Both demographic and disease-specific baseline information were described using frequencies and percentages for categorical variables. Ordinal variables were described as medians and interquartile range (IQR). Interval variables were described as group means and standard deviations (SD). The study received ethical exemption by the Aga Khan University Hospital Ethical Review Committee (AKUH ERC) before data collection and analysis were done.

## Results

A total of 111 patients were included in the study, from a population of 4925 patients who underwent surgery in our time frame, with males constituting 74.8% and females 25.2% of the cohort. The median age of patients was 36 years with an interquartile range of 33. Table 1 summarizes the demographics and their relation to in-hospital mortality.

**Table 1:** Patient demographics compared with mortality (n=111) ASA: American Society of Anesthesiologists Physical Status Classification System

Demographics			In-hospital mortality (n=14)	Level of significance (p-value)
Sex	Male	83 (74.8%)	10 (9.0%)	0.748
	Female	28 (25.2%)	4 (3.6%)	
Seizures		12 (6.5%)	1 (0.9%)	>0.99
ASA level	1	0 (0%)	0 (0%)	0.179
	2	45 (40.5%)	3 (2.7%)	
	3	43 (38.7%)	7 (6.3%)	
	4	21 (18.9%)	3 (2.7%)	
	5	2 (1.8%)	1 (0.9%)	
Deranged coagulation profile		24 (21.6%)	3 (2.7%)	>0.99
Admission status on redo craniotomy	Elective	33 (29.7%)	3 (2.7%)	0.549
	Emergent	78 (70.3%)	11 (9.9%)	
Age (years), mean		36 (SD = 1-84)		

**Figure 1:**
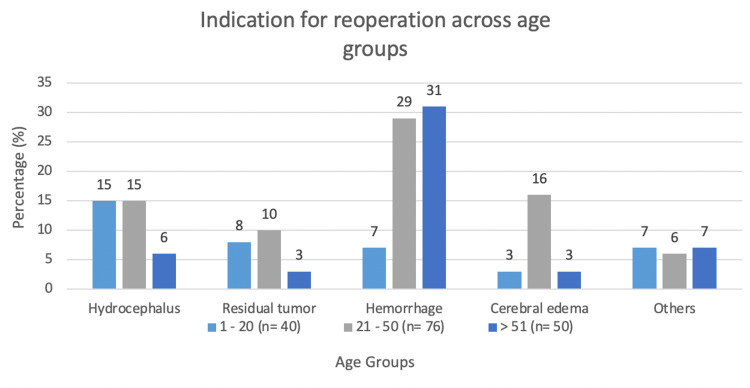
Indication of reoperation across age groups

**Figure 2:**
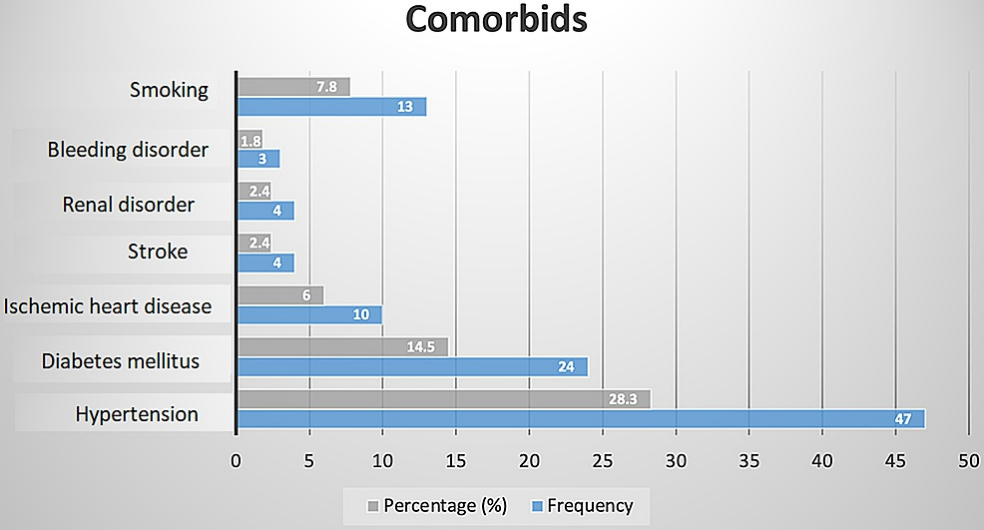
The percentage and count for patients with a history of pre-existing disease and smoking

The most common indication for the primary surgery was tumor resection (48%), followed by hemorrhage (17%), trauma (15%), vascular insult (12%), and miscellaneous causes (8%). Figure 1 highlights the most major indications across age groups. Unplanned reoperation was most commonly due to hemorrhage (40%), with other causes being hydrocephalus (27%), cerebral edema (13%), residual tumor (in cases of partial resection in the initial surgery) (13%), and miscellaneous (12%) (examples include neurological deficit and residual tumor). In hemorrhage, the most common subtype was subdural (17%), epidural (12%), and intraparenchymal (11%). More than 50% of the patients presenting with hemorrhage post-craniotomy were hypertensive (p=0.014). Figure 2 summarizes the other comorbidities among our cohort. 

**Figure 3:**
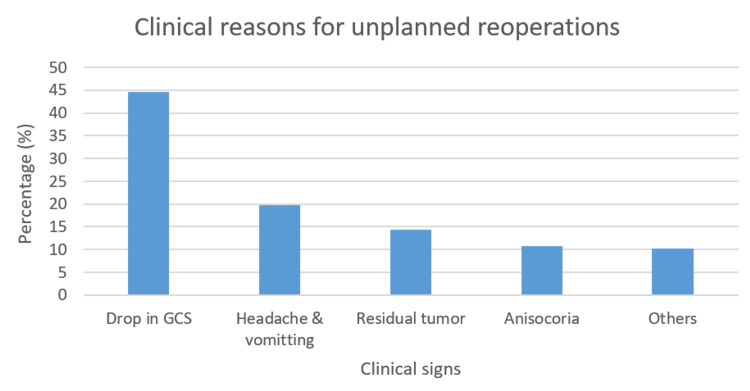
Reasons for unplanned operations GCS: Glasgow Coma Scale

The patients on antiplatelet and anticoagulant regimens were at a higher risk of being reoperated, especially in patients presenting with traumatic brain injury. Approximately, 7% of the patients presented with thrombocytopenia out of which 81.9% suffered hemorrhage. Among 111 patients in the study, 4% had deranged activated partial thromboplastin time (aPTT) and 4% had deranged prothrombin time (PT). Table 2 summarizes the blood loss in the index and redo surgeries as well as the mean time between craniotomies. In the instance of multiple presenting complaints, the one with the most impact on quality of life was used for analysis. The clinical reasons for unplanned reoperation are summed up in Figure 3.

**Table 2:** Intraoperative characteristics

Intraoperative characteristics		
Blood loss (mL), median, and range	Index craniotomy	300 (0-3000)
	Redo craniotomy	200 (0-4350)
	Time between the index and redo craniotomy, median (days)	5 (0-1095)

Tumor resection comprised a significant portion of the initial craniotomies (48.2%), with tumor location a significant predictor for reoperation (p<0.05). Table 3 summarizes our findings.

**Table 3:** Location of tumor and its complication CP: cerebellopontine

Location of tumor (% of tumors)	Complication (% in each category)
Supratentorial (35%)	Hemorrhage (40%)
Posterior fossa and CP angle (30%)	Hydrocephalus (37.5%)
Intraventricular (17.5%)	Hydrocephalus (50%)
Skull base (17.5%)	Cerebral edema (50%)

Among those who returned to the operating room, 30% had a planned elective surgery and 70% had an emergent (unplanned) return to the operating room. Both had a similar operating time with no significant difference in time spent during surgery. There was also no difference in blood loss, or indication for surgery (symptomatically) between redo emergency and elective cases. However, there was a significant difference between the two groups in terms of time between the index and redo craniotomies (p=0.04). The mean time between the two surgeries (index surgery and reoperation) was shorter in emergent cases with an average of only five days as compared to a mean of 15 days in planned re-operative cases.

While evaluating the time interval between initial and unplanned reoperation, the data were categorized into three categories: those operated within 24 hours (n=33, 20%), within one to seven days (n=66, 40%), and from eight to 30 days (n=67, 40%). Indication for reoperation was further analyzed. For patients reoperated within 24 hours, the leading indicator was extradural hemorrhage (n= 13, 39.3%) followed by cerebral edema (n=9, 27.2%), as evidenced on imaging, and intraparenchymal hemorrhage (n=5, 15%). The mortality rate in this group was 36%. For patients reoperated within one to seven days, the leading indicator was hydrocephalus (n= 17, 25.7%), subdural hemorrhage (n= 12, 18.1%) and cerebral edema (n=10, 15.1%). The mortality rate in this group was n= 38, 56%. For patients reoperated between eight and 30 days, the leading indicator was hydrocephalus (n= 18, 26.8%), subdural hemorrhage (n= 15, 22.3%) and residual tumor (n= 10, 14.9%). The mortality rate in this group was n=5, 8%. Table 4 portrays post-operative characteristics after redo craniotomy compared with in-hospital mortality. 

**Table 4:** Post-operative characteristics compared with mortality (n=111) ICU: intensive care unit

Post redo craniotomy symptoms		In-hospital mortality	Level of significance (p-value)
New neurological deficits	12 (10.8%)	3 (2.7%)	0.211
Worsening of old deficits	18 (16.4%)	10 (9.1%)	0.000
Surgical site infection	10 (9%)	2 (1.8%)	0.612
Shunt failure	3 (2.7%)	1 (0.9%)	0.335
Cerebral edema	8 (7.2%)	3 (2.7%)	0.062
Abscess	5 (4.5%)	0	>0.99
Meningitis	7 (6.3%)	2 (1.8%)	0.214
Bleeding/hematoma	3 (2.7%)	1 (0.9%)	0.335
ICU stay, median	2 (0-30)		

**Figure 4:**
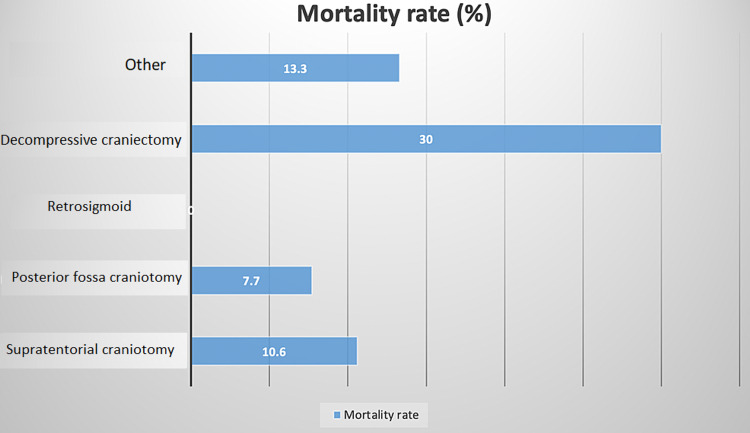
Mortality rate in different types of craniotomies

In-hospital mortality was compared between elective cases and emergency cases with the emergent cases (on initial presentation) having a higher rate of 19% vs 7%. However, the difference was not statistically significant (p>0.05) between the two groups. Mortality rate between types of craniotomies is shown in Figure 4, retro-sigmoid approaches yielded no mortalities.

**Figure 5:**
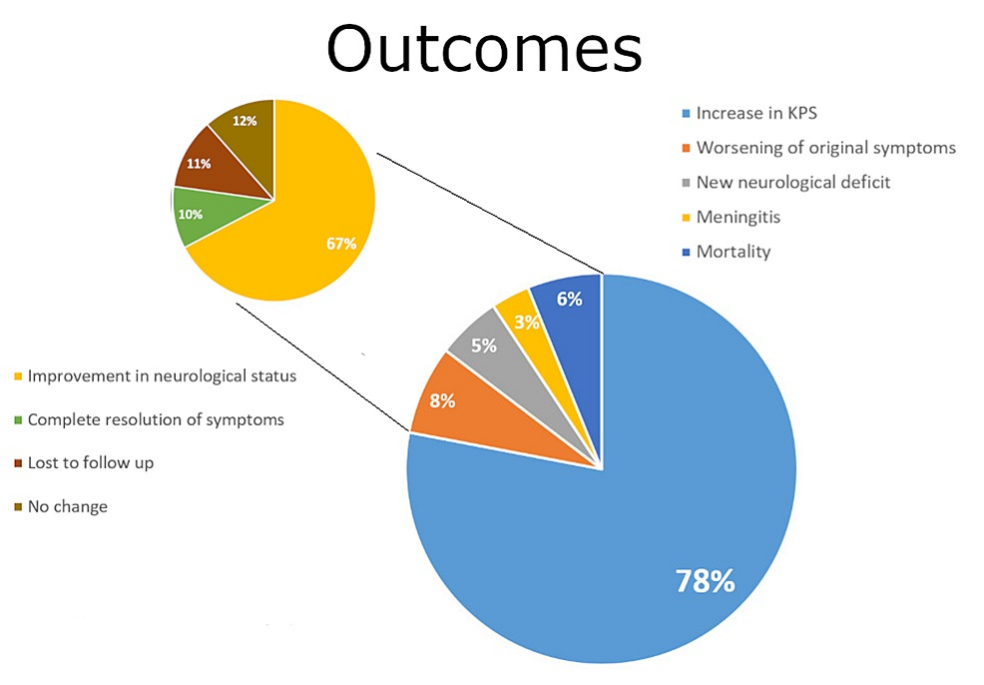
Outcomes of reoperation KPS: Karnofsky Performance Score

Outcome after unplanned reoperation is of particular interest. Seventy-eight percent of patients experienced positive outcomes (increased KPS) with improved neurological statuses. From the remaining 22% (with reduced post-op KPS), around 18% experienced worsening of the original symptoms, 13% developed additional neurological deficits, 7.8% developed meningitis and there was a mortality rate of 15%. At later follow-up, 67.3% of patients had further improvement of neurological status and 9.9% experienced complete resolution of symptoms. The lost to follow-up patients comprised 11.3% of our cohort. Figure 5 summarizes these results. 

## Discussion

Reoperation rates depend on risk factors such as co-morbidities, original insult, and neurological status. Figure 1 shows that the major indicator for each age group was different in our cohort. Among patients between the ages of one to 20 years, hydrocephalus was the most common reason for reoperation whereas in the 21-50 and >51 years age groups, it was hemorrhage. Patients with hypertension and thrombocytopenia were at a greater risk of hemorrhage after craniotomy and correspondingly had higher rates of reoperation. The most prominent reason for reoperation overall was decreasing GCS (44%), followed by headache and vomiting (20%) which indicated an increased intracranial pressure (confirmed via lumbar puncture). The development of new neurological deficits or worsening of the original insult was more common in patients who underwent reoperation within the first week of the index surgery. Overall return to the operating room for emergency craniotomy is also highest closer to the original date of surgery (mean five days). We can extrapolate from our data that this is likely due to more severe complications in these cases, which need immediate intervention. This is reflected in several studies for both neurosurgery and general surgery [8,9]. In our cohort, the most common indication for redo emergency surgery was hydrocephalus followed closely by cerebral edema.

Readmission within 30 days for cranial neurosurgical service has been studied by Moghavem et al., and their analysis showed that readmission was highest for the patient initially admitted for neurovascular insult [10]. Zheng et al. reported a reoperation rate of 51.5% due to post-operative hemorrhage at their center in China while other studies from France, the United States of America, and Russia showed a range from 4.2% to 31.5% at their centers [11-15]. Our data showed that patients with trauma with hydrocephalus were most likely to undergo reoperation, with no specific primary surgical indication being significantly higher.

McLaughlin et al. reported a quality assessment regarding reoperations and their preventability over a 42-month period, within two major medical centers in the United States of America [9]. Reoperations that occurred within seven days of the index surgery were reviewed and the overall incidence was found to be 2.6% compared to our incidence of 2.25%. Most patients who underwent early unplanned reoperation initially had surgery for shunt-related conditions (34.4%) or intracranial tumor (23.5%) whereas in our case, hemorrhage was the most common.

Zattra et al. compared morbidity and mortality between patients from Switzerland and Italy undergoing redo craniotomy vs those without redo craniotomy [16]. Morbidity was defined as a significant drop in the Karnofsky Performance Score (KPS). Overall, the risk for morbidity was not significantly increased after repeated craniotomy and microsurgery for tumor removal. Similarly, the rate of mortality and severe complications was not significantly different between patients with redo operations vs those with only a primary surgery. Notably, these patients presented with brain tumors and underwent oncological surgeries, as compared to our sample which has a wider range of pathologies at index surgery. Another study by Chen et al. in Singapore showed better survival after redo craniotomy in glioblastomas over a 10-year period; however, their time to redo craniotomy was not defined by a specific cutoff [17].

Reoperation is also dependent on surgeon preference and has a lower interrater reliability and need for surgery score as compared to first-time surgery [18]. Ramayya et al. conducted a prospective review (in the United States of America) to correlate surgical decision-making in neurosurgery with redo surgery as one of the primary cohorts. There was significantly less consensus amongst attending neurosurgeons regarding redo craniotomy than primary craniotomy which causes variability in surgical care and reoperation rate [18]. In our study, we did not assess the attending neurosurgeon decision pathway but the authors hope to pursue it at a future junction. There has been some investigation into the feasibility of surgical adverse event prediction to reduce the unplanned return to the operating theatre (UROT); Marini et al. demonstrated improvement in their morbidity and mortality conference (MMC) when linking UROT to MMC which highlight non-conformation care processes and subsequent room for improvement in France [12]. In the pediatric population, Mukerji et al. report a 17% 30-day reoperation rate at their center in the United Kingdom with 44% of all operations were related to CSF diversion [19]. Similarly, when looking at a 48-hour reoperation rate, CSF shunt placement and hydrocephalus were the major indications (40.8%) reported by Roy et al., which is reflected in our patients as well [20].

Algattas et al. conducted a study in the United States of America to identify clinical factors predictive of patients returning to the OR for hemorrhage after craniotomy [21]. The major indicator for unplanned reoperation was hemorrhage (63.8%) which is higher than our rate of reoperation due to hemorrhage (40%). Pre-existing hypertension and a history of bleeding disorder were significant factors reported by Algattas et al. which were associated with a return to the operating room.

Chen et al. conducted a study in Singapore demonstrating increased median survival and KPS for patients undergoing redo craniotomy as opposed to purely pharmaceutical treatment [17]. Our study also showed a comparative increase in neurological status in our patients, with mixed index surgery indications, after redo craniotomy. This points towards a tangible benefit in aggressive reoperation, especially for higher grade tumors. Troya-Castilla et al. also investigated patients with brain tumors in Spain to identify the benefit of aggressive resection through early reoperation [22]. Overall survival and progression-free survival of 58 patients was studied. Reoperation achieved complete tumor resection in 58.62% of all reoperated patients. While functional prognosis was similar between groups that had reoperation and those that did not, overall survival and progression-free survival were higher in patients that underwent the operation. This is also reflected in our results although we had not distinguished between patients undergoing purely oncological surgeries or surgeries for other indications such as trauma.

Similar to our study, Molina et al. also conducted a study to analyze the reoperation rate in their center in Germany with the aim of assessing it as an indicator for quality in neurosurgery [23]. A total of 3760 patients were included in the study over a period of two years, with 370 patients undergoing reoperation within 30 days, and 193 patients out of those undergoing unplanned reoperation within seven days of the index surgery. As with our findings, the most common indication for reoperation was hemorrhage (n=107), closely followed by external drainage-associated infections or dislocation (n=105).

Our study had a total of 4925 patients with a reoperation rate of 2.25%. Table 5 shows a comparison of reoperation rate in other studies. The most common reason for index surgery in our dataset was tumor resection (58%), which was consistent with other studies. Hemorrhage and hydrocephalus were indications for redo craniotomy, in comparison with other studies where indications for redo craniotomy were surgical site infection and shunt failure. The most common clinical reasons for unplanned reoperations included drop-in GCS, headache and vomiting, residual tumor, and anisocoria.

**Table 5:** Comparison of reoperation studies over the years

Study/year	n	Reoperation rate	Indication for index surgery	Indication for unplanned reoperation	Mean no. of days between index and reoperation
McLaughlin et al., 2015 [9]	6912	2.60%	Shunt related (34%)	Shunt failure (28%)	3.0 ± 1.9
			Intracranial tumor (23%)	Post-operative bleeding (20%)	
Algattas et al., 2016 [21]	5520	1.50%	Tumor excision (50.6%)	Hemorrhage (63.8%)	6.0 ± 6.9
Dasenbrock et al., 2017 [2]	11,462	3.10%	Tumor resection (NA)	Hemorrhage (22.5%)	NA
				Surgical site infections (11.9%)	
This study	4925	2.25%	Tumor resection (58%)	Hemorrhage (40%)	8.7 ± 8.5
				Hydrocephalus (22%)	

Our study had several limitations. A longer follow-up time could have better-predicted outcomes of redo craniotomy in our study. Moreover, several patients (11.3%) were lost to follow-up. Since our study was restricted to one of the three JCI accredited tertiary care centers in the country with good nursing care, the rate of reoperation might not be representative of other hospitals in the region. Additionally, the heterogeneity of the patient collective will vary significantly based on the level of the hospital and regional characteristics. In the future, studies assessing reoperation rate could better assess the reoperation by increasing follow-up time, and by including more centers. Lastly, since tumor resection is the most common reason for index surgeries, studies can solely focus on the reoperation rate of brain tumor cases.

## Conclusions

Assessing the quality of care is an important metric in healthcare, especially where standards are often compromised due to limited resources and personnel. Reoperation rate is particularly valuable in surgical specialties as it often points towards systematic problems rather than issues at just one level. Our center’s reoperation rate was consistent with the average range among other centers globally at 2.25%. Patients in our population need to be closely monitored for hemorrhage in the short term (one to two days) and hydrocephalus in the long term (two to 30 days) to intervene early if needed. Our study demonstrates that antiplatelet medication, anticoagulation medication, and hypertension are common risk factors for reoperation. Special care needs to be addressed to these patients to aggressively prevent the need for further surgeries, and thus improve long-term morbidity and mortality.
